# Supplementary material for: Perceived Health Benefits in Vestibular Schwannoma Patients with Long-Term Postoperative Headache: Insights from Personality Traits and Pain Coping—A Cross-Sectional Study
Source: J Pers Med. 2024 Jan 8;14(1):75. doi: 10.3390/jpm14010075 (PMC10817612; doi:10.3390/jpm14010075)
Supplement: Supplementary file 1 [file jpm-14-00075-s001.zip › jpm-2787651-supplementary.pdf]

Table S1. Comprehensive correlation matrix for personality, psychological burden and perceived health benefit as well as demographic and disease-related variables (n = 50).

	FESV									*HADS-D*						*GBI*			
	HD	ANX	ANG	APC	CR	EC	MD	CA	RR	depression	anxiety	total	hearing loss	facial paresis	pain (NAS)	general	social support	physical health	total
gender	-.08	-.04	-.15	-.13	.17	.17	.01	-.24	.23	-.28	-.05	-.16	-.05	.23	.03	.12	-.06	.26	.24
age	-.27	-.32*	-.32*	.08	.27	.30*	.18	-.23	.22	-.18	-.26	-.23	-.31*	.05	-.20	-.02	.30	.25	.20
hearing loss	.16	.29*	.37**	-.13	-.33*	-.19	-.17	.11	.01	.37**	.04	.20	-	-	-	.01	-.16	-.06	-.12
facial paresis	-.03	.01	-.23	.14	.06	.02	-.11	-.13	-.16	-.13	.09	-.02	-	-	-	.17	-.07	-.07	.18
pain (NAS)	.24	.21	.19	.11	.01	-.10	-.23	-.17	.15	.25	.02	.09	-	-	-	-.23	-.08	.18	-.28
TIPI-G																			
extraversion	-.21	-.32*	-.39*	.09	.23	.30*	-.01	.12	-.06	-.49**	-.23	-.34*	-.29*	-.03	-.39**	.07	.14	.33*	.20
agreeableness	-.02	-.03	-.13	.25	-.02	.19	.05	-.24	.18	-.07	-.12	-.10	-.01	.17	-.003	.19	.14	.21	.23
conscientious ness	.10	-.03	.04	-.07	.16	.15	.17	.14	.27	-.06	-.31*	-.24	-.11	-.01	.06	-.14	-.00	-.20	-.21
emotional stability	-.42**	-.58**	-.59**	.13	.20	.48**	.01	.02	.09	-.35*	-.63**	-.56**	-.15	-.17	-.31*	.02	.07	.20	-.14
openness	-.46**	-.50**	-.42**	.24	.15	.23	-.12	.12	.05	-.47**	-.29	-.35*	-.17	-.13	-.32*	.11	-.12	.17	.14
GBI																			
general	-.40**	-.35*	-.25	.39**	.40**	.39**	.03	.18	.08	-.25	-.24	-.29	.01	.17	-.23	-	-	-	-
social support	.05	-.06	-.02	.04	.02	.07	-.11	-.14	.06	-.01	.09	.04	-.16	-.07	-.08	-	-	-	-
physical health	-.45**	-.30*	-.25	.18	.33*	.46**	.09	.10	.14	-.40*	-.33*	-.37*	-.06	-.07	-.10	-	-	-	-
total	-.44**	-.37*	-.32*	.31*	.49**	.50**	.13	.20	.10	-.39*	-.27	-.38**	-.12	.15	-.28	-	-	-	-

Abbreviations. TIPI-G = Ten Item Personality Inventory German, FESV = Questionnaire for the Assessment of Pain-related Behavior, NAS = numerical analogue scale, HD = helplessness and depression, ANX = anxiety, ANG = anger, APC = action-planning competence, CR = cognitive restructuring, EC = experience of competence, MD = mental distraction, CA = counteractive activities, RR = rest and relaxation, GBI = Global Benefit Inventory. * $p < 0.05$, ** $p < 0.001$.
